# Silences and Omissions in Reporting Epidemics in Russian and Soviet Prisons, 1890-2021

**DOI:** 10.1093/jhmas/jrad047

**Published:** 2023-08-31

**Authors:** Mikhail Nakonechnyi, Judith Pallot

**Affiliations:** University of Helsinki, Finland; University of Helsinki, Finland; University of Oxford, UK

**Keywords:** Russia, Prisons, epidemics, public communication, typhus, cholera, MDR-TB, Gulag, COVID-19

## Abstract

Penitentiary systems serve as breeding grounds for all kinds of diseases. Drawing upon new archival materials, this article examines the history of the management and reporting of epidemics in the Russian prison system from the late Imperial period to the present day. We use the case studies of cholera (1892-1893), typhus (1932-1933), and pulmonary tuberculosis (the 1990s) to examine how the general political and social conjuncture at different times affected the response of prison authorities to epidemics to show that, notwithstanding major shifts in society and polity, there was continuity in the management of epidemics by prison authorities in the long twentieth century. However, there were fundamental discrepancies in the way late Imperial, Soviet, and post-Soviet Russia reported epidemiological emergencies in prisons. We argue that Russia’s tumultuous past has reinforced the tendency among the Russian penal administration towards a lack of transparency that has persisted to the present day, in relation to the latest, COVID-19, epidemic.

## Introduction: Prison Epidemics in the Context of Russia’s Turbulent History

In March 1909, Stepan Stepanovich Khrulev, director of the Main Prison Administration of the Imperial Ministry of Justice, stood before the State Duma, the incipient Russian parliament, to reassure that his ill-reputed department was doing everything to tackle the devastating typhus epidemic spreading rapidly through the Empire’s prisons.^1^ A year later Khrulev ascended the rostrum again and reported to the skeptical deputies, “…I can tell you…that roughly a year ago the number of typhus patients in prisons of the entire Empire reached 3,000; now, it plummeted by ten times, and does not exceed 300-400, and was hovering around 130 in the autumn.”^2^ In historical hindsight, the remarkable feature of these appearances before the parliament was not the putative success of measures taken that ran counter to the ubiquitous grim portrayals of the prison system in the media, but rather Khrulev’s apparent readiness to raise the subject in a public forum to provide excuses. Crucially, Khrulev’s argumentation was bolstered by medical data, openly published by the government and available to civil society for critical analysis.

Within twenty years, and as archives opened since 1991 have laid bare, typhus again wrought havoc in the penal establishments of the young Soviet state, particularly in a new concentration camp located on the Solovet’skii archipelago in the White Sea. However, Aleksandr Petrovich Nogtev, the commandant of Solovet’skii camp, did not feel compelled to provide any sickness and mortality statistics to the public, make excuses before the All-Union Congress of Soviets, or even appear there at all. Local newspapers ceased to be outraged about the maladministration of epidemiological emergencies in prisons as in late Imperial times. In fact, they stopped reporting on epidemics in the penal system entirely. Opponents and admirers of the Soviet regime lost access to any prison medical data as well as the possibility of publicly arguing about it. Even the mere fact of epidemics behind bars became a piece of highly classified information. The public debate about the issue essentially froze. The extraordinary turnaround in the epidemic reporting in a short period is explained by the Revolution of 1917 in Russia that led to the foundation of a novel type of polity in which bad news was rigorously kept from the public domain to a degree yet unknown in Russian history. In the decades that followed there have been similar reversals in news management about health and mortality among vulnerable populations associated with major societal change. These continue to the present day, most recently in relation to the ongoing COVID-19 pandemic.

In this article, we explore the history of epidemics in Russian prisons during the long twentieth century, focusing on how the reporting of epidemics changed over time. Russia offers an instructive case for exploring how states seek to manipulate understandings of the threat that prisons pose to public health. Firstly, over a 130-year period, the country experienced radical shifts in governance that changed the political, economic, and social context in which punishment and medical services were administered. Reflecting these discontinuities, punishment modalities in Russia also underwent fundamental transformations. In the last decades of the nineteenth century, Imperial Russia turned away from penal transportation and internal exile towards the modern penitentiary. But the “birth of the prison” was brought to a halt in the early twentieth century.^3^ The Bolshevik Revolution of 1917 discarded the modern idea of individualized punishment and cellular confinement for the principles of collectivism and communal labor as the appropriate vehicles for prisoner rehabilitation under socialism. These principles were soon to be realized, but distorted, in the forced labor camps and deportations of the Stalinist repressions.^4^ Traces of the purportedly “socialist” principles for correcting offending behaviors, social deviancy, and political opposition remain in Russia’s penitentiary system to the present time. Russia still transports convicted offenders to the geographical peripheries, accommodating them in dormitory blocks in correctional colonies, a system that has been described as “in exile imprisonment.” Historically, Russia’s system of punishment has had a profound effect on its ability to contain epidemics.^5^ Given the drastic ruptures of Russian history, it is yet not entirely clear how the knowledge transfer pertaining to the management of epidemiological emergencies occurred across consecutive generations of penal officials over a 130-year timeframe. In this article, we contend that the revolutions of 1917 marked a sharp break in the way the state informed the public and professional medical community about epidemics in prisons. Nonetheless, continuities existed on the practical level of handling epidemics as pre-revolutionary doctors and paramedics continued to serve in the same capacity in the Soviet penal system (often being prisoners themselves).

Secondly, the Soviet Gulag was one of the harshest and inhumane prison regimes in global history and, justifiably, it is frequently compared with the concentration camps of Nazi Germany.^6^ The USSR did not have the equivalent of the extermination camps, although this point is disputed by some Gulag scholars.^7^ The function of camps in the USSR was to mobilize labor for national economic construction and it was primarily for this reason that numbingly large numbers of mostly innocent people were dispatched to the resource frontier and construction sites across the USSR to serve long sentences under conditions of the most extreme cruelty and harshness. The Gulag offers a case study of the management of epidemics in the most total of total institutions, where medical staff were judged by their success in reproducing a labor force equal to meeting their camp’s production targets.

Thirdly, Russia provides an example of the challenges that all prison systems face in news management. Cultural historian Philip Smith has observed that the treatment of prisoners touches on deeply embedded cultural sensibilities.^8^ Following anthropologist Mary Douglas, Smith argues that control of spiritual impurity, evil, dirt, and disgust that Douglas understood as a form of social disorder is a universal human project. Prisons, by their very nature, concentrate these disorders and they are deemed to fail if their effect is to increase the sum total of pollution and contagion in society, rather than control and contain them.^9^ Historically, Smith argues, transformations in how societies punish people owe less to the rational arguments of penal reformers than to the dialogue that develops between government and society around deep-seated cultural norms about physical and spiritual purity. In Smith’s view, maintaining control over that dialogue, which if lost can lead to disruptive calls for reform, is a primary aim of prison authorities in news management.^10^ Despite Russian prison authorities at various times requiring prisoners to sign non-disclosure agreements, news of epidemics leaked into the public domain as hundreds of thousands of prisoners moved through the “revolving door” between prison and society.^11^ Prison governors, ever wary of negative reports reaching their superiors have, at all times, had to remain alert to seemingly incorrect decoding of their messages about prisoners’ health.

We need to sound the usual note of caution about anachronism in relation to our case studies. We have taken care not to project backwards to the nineteenth and early twentieth centuries current sensibilities and expectations about the management of epidemics. We also have considered the state of scientific knowledge and level of medical, hygienic, and technological development in Russia in the periods we study. As late as the 1950s, smallpox, scarlet and yellow fevers, measles, erysipelas, cholera, whooping cough, diphtheria, and other infectious maladies (or zymotic diseases, as they were called at the time) occasionally reached flagrant proportions in prison systems across the globe. The death rates of prisoners during these epidemics were exceptionally high even by the standards of the time. Cholera, for instance, if left untreated, could lead to death within two days with 50% probability. The advent of sulfanilamide drugs and penicillin in the 1940s was hailed as a structural game changer, marking a shift from the use of preventive to therapeutic methods of combatting epidemics. Advances in public health and the introduction of the infrastructures for running water and modern sanitary systems were also credited with making a major contribution to the elimination of the principal nineteenth-century killers of prisoners, such as cholera, in jurisdictions in economically developed countries.

Advances in scientific knowledge of the etiology and pathology of infectious diseases have questioned nineteenth- and twentieth-century ideas about the bacteriological revolution that underpinned the new therapies used to control epidemics in the developed countries, whilst time was to reveal the negative consequences of the overuse of antibiotics.^12^ However, the potential benefits that did accrue from new approaches to epidemic management in prisons in Western jurisdictions were often delayed in Russia and the USSR. Prison medicine suffered from a double isolation as a result of the Soviet state’s isolation from western science and of prison health service’s isolation from medical advances that were introduced into the country’s civilian health services. There was a huge gap, for example, between the health needs of the Soviet population and the production of penicillin after World War II, which was a consequence of the slow development of the USSR’s domestic penicillin production and the prohibition of its importation from the west, as a consequence of the Cold War.^13^ Inevitably, prisoners were a low priority in the USSR for treating with any new therapy making its way into civilian clinics. The geographical dispersal of the prison estate which subjected prisoners to arduous journeys in overcrowded rail carriages and the practice of accommodating prisoners in communal dormitories at their destination ensured that infectious diseases thrived and continued to spread through the penal system. This problem remains to the present day.^14^ Although major advances were made compared with previous decades in the fight against typhus, cholera, and dysentery, the vast majority of acute infectious illnesses that afflicted prison populations remained incurable in Russian prisons into the second half of the twentieth century and resulted in a continued reliance on basic preventative measures, such as quarantine.

## Prior Historical Research on Epidemics in Russian and Soviet Prisons

The history of prison medicine discussed in western literature is only partly relevant to the situation in Russia. A literature on prison medicine in Imperial Russia exists, but is rather sparse. There are several informative, empirical studies written by pre-revolutionary prison doctors that describe some of the epidemics that ravaged prisons.^15^ There are also published reports of external inspectors before 1917, which could be overtly critical. A case in point is a rather uncomplimentary and sober overview of prison sanitation published in the official *Tiuremnii Vestnik* (*The Prison Herald*) by military physician Gur’iev, who was sent to inspect prisons in several Imperial provinces during the devastating typhus epidemic of 1907-1910.^16^

After the 1917 Revolution, Soviet historians, driven by the ideological axiom that Tsarist prisons were the deadliest in the entire world, neglected pre-revolutionary prison medicine. Thus, the classic *History of the Tsarist Prisons* by Mikhail Nikolaevich Gernet accused Old Regime’s prison doctors of premeditated extermination of convicts but offered almost no health data to corroborate this bold claim.^17^ After the collapse of the Soviet Union in 1991, Tsarist prison medicine and its relationship with epidemics were confined to the margins of academic research.

In western literature on punishment in Russia, health has surfaced as a peripheral issue in the histories of exile and imprisonment in late Imperial Russia.^18^ The calculations made by Stephen Wheatcroft of fluctuations in morbidity and mortality rates in 1880-1917 are a notable exception. He argued that before 1905-1906, there was indeed “remarkable progress in Russian prison reform and prison conditions” and “great improvement in [prisoner] health and welfare...”^19^ He discovered a major upswing in mortality rates in the turbulent years of 1907-1912, although he overlooked the contribution of the typhus and typhoid epidemics at that time. For the period of the Revolution and Civil War, Mary Schaeffer Conroy has used recently opened archives to describe the professionalism and dedication of the prison doctors who struggled to maintain prisoners’ health at this time and to prevent the spread of infectious disease to the population at large.^20^

There is more research on healthcare in Soviet-era prisons, mainly focusing on the period 1930-1953, spanning the Stalinist repressions. The pioneering studies of Boris Nakhapetov, Oxana Ermolaeva, Golfo Alexopoulos, and Dan Healey have created a firm empirical foundation for further study of the institutional history of the Gulag medicine, epitomised in the so-called Sanitary department (SANO), an embedded health service.^21^ According to Healey, in 1938 the Gulag, “employed 1,830 qualified doctors, of whom perhaps one-third were prisoners. At the same time, 7,556 nurses and paramedics worked in the system, and a large proportion of these were prisoners.”^22^ Some had pre-revolutionary professional medical education.^23^ However, the question of epidemics and the measures to contain them remain under-researched. This literature is largely preoccupied with mortality rates among the Gulag’s victims and the question of whether prisoners were used for medical experimentation.^24^ Prior to the USSR’s collapse in 1991, researchers had to rely exclusively upon secondary data and guesswork, informed by testimonies of Gulag survivors, to estimate mortality. Improved access to archives in the 1990s led to downward revision of the highest estimates, and today a total estimate of excess mortality varies from six million to 2.5 million as against the official figure of 1.7 million.^25^ This figure includes epidemic deaths, although the primary cause of excess deaths was starvation, especially during crisis years of famine and WWII.

The prisons of the late-Soviet era are poorly researched compared with the Gulag, but in this period the focus shifts to the use of psychiatric detention against regime opponents.^26^ As for contemporary post-Soviet prison medicine and epidemics, the literature is dominated by so-called departmental (*vedomstvennaia*) historiography, produced by scholars, mostly jurists, affiliated with Federal Prison Service.^27^ This scholarship is tendentious and lacks transparency. However, considerably more impartial and critical work on contemporary prison medicine and practitioners in Russia is produced in the field of sociology.^28^ Nonetheless, none has yet looked into the management and reporting of epidemics in Imperial prisons that we discuss below.

## The Cholera Epidemic in Russian Prisons, 1892-1893

The Russian Empire had been incrementally improving its public health in the late 1800s and in this respect, it was no different from other European countries. It had a poorly developed health infrastructure, even by the modest standards of the nineteenth century. In her detailed history of cholera in the Volga port town of Saratov from 1823 to the onset of the First World War, Charlotte Henze produces a micro portrait of the medical and social disasters associated with successive epidemics that can be generalized to the whole country.^29^ Progress towards improvement in the sphere of public health was hampered by the size of the empire, negligible investment in sanitary reforms, and a large uneducated peasant population wedded to folk medicine. The general population was suspicious of the medical profession, as was demonstrated by the murder of some doctors by superstitious peasants in cholera riots. The late nineteenth-century empire was characterized by elevated infant mortality rates, low life expectancy, and a high incidence of infectious disease. Devastating epidemics periodically ravaged the country throughout the nineteenth and early twentieth centuries, leading to hundreds of thousands of excess deaths among the civilian population, the cholera outbreak of 1848 a particularly egregious example.^30^ Even members of the Romanov dynasty succumbed: future emperor Alexander III had typhoid in 1866; Empress Maria Alexandrovna, typhoid; Grand Duchess Ksenia Alexandrovna, typhoid in 1888; Emperor Nicholas II, a severe case of typhoid in 1900; and Grand Duchess Tatiana, typhoid in 1913.^31^

The 1892-1893 cholera epidemic in prisons was an episode in the fifth serious outbreak of cholera in nineteenth-century Russia that occurred between 1891 and 1896.^32^ The first two years of the outbreak coincided with the most ill-famed of the late Imperial famines in the southeast of the Empire when deaths among the weakened population reached at least 342,574, certainly an understated number.^33^ The pioneering work of British epidemiologist John Snow in the 1850s and German microbiologist Robert Koch in the 1890s brought a greater understanding of cholera bacteria and the etiology of the disease to Russia and was important in reducing death rates.^34^ Although by the 1890s Russia had embarked on penal reform, prisons became epicenters for disease because of the high turnover rate of prisoners linked to the continued use of exile. After a short stay in prisons, offenders sentenced to exile and hard labor spread disease to the peripheries of the largest landlocked Empire in the world. Every year, roughly 10-15 thousand exiles and prisoners travelled under armed guard on foot, and later by trains, ships, and barges, to inhospitable fringes of the Empire - the Urals, Siberia, and the Far East.^35^

The Imperial prison authorities readjusted the system of inmate relocation (*etapirovanie*) but not in time to contain the spread of the disease. The first cholera case in the prison system occurred in June 1892 in Astrakhan at the mouth of the River Volga in southern Russia. The region’s governor petitioned the Imperial Main Prison Administration in St. Petersburg (hereafter GTU for *Glavnoe Tiuremnoe Upravlenie*) to halt all prisoner transportations to prevent the disease spreading into Turkestan and along the coast of the Caspian Sea. The central authorities responded by halting the forwarding of prisoners originating in the Caucasus and provinces on the Volga River to exile destinations. Acknowledging the potential of prisons and exile to infect the free population, Ivan Nikolaevich Durnovo, the Minister of Internal Affairs, sent Mikhail Nikolaevich Galkin-Vrasskoi, the GTU director, to the lower Volga region to personally coordinate relief efforts.^36^ Meanwhile, cholera appeared on 4 July in prison of the town Perm’ in the foothills of the Urals, which was one of the stopping points on the exile routes east to Siberia. Reports of outbreaks of cholera in other prisons quickly followed, including in Rostov-on-Don in the north Caucasus on 18 July, and Tobolsk in Siberia at the end of July. Moscow Transit Prison (*Moskovskaia peresyl’naia tiur’ma*), a special type of institution tasked with prisoners’ conveyances, was a crucial logistical hub in the exile transportation system, and to protect it from contamination the GTU prohibited inward movement of prisoners and exile parties to it from its feeder regions in central European Russia.

Regional authorities took their own actions at the same time as the capital. When cholera appeared in Taganrog on the Black Sea coast, the regional governor halted the movement of prisoners from the coastal port towns of Kerch, Mariupol, Berdiansk, and Eisk. In Astrakhan, where the first case of a prisoner contracting cholera had been recorded, further cases were prevented by the speedy introduction of total quarantine in the prison. These measures were successful and by August 1892, the epidemic among the prison population started to recede. On 10 October 1892, Galkin-Vrasskoi allowed the resumption of all prisoner transports, except from the Caucuses where cholera was still prevalent. Although the illness reappeared in prisons in 1893, it never reached the levels of 1892.^37^

**Figure 1.**
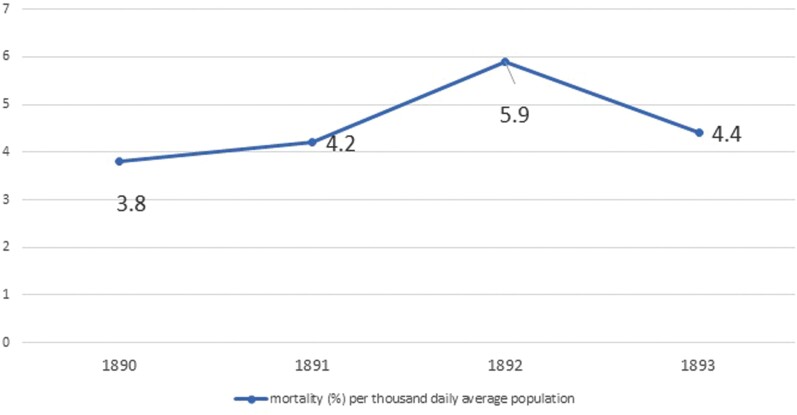
Mortality in Imperial Russian prisons, 1890-1893 Source: Calculated based on *Otchet po glavnomu tiuremnomu upravleniu za 1890,1891,1892,1893*. Wheatcroft, “Crisis of the Late Tsarist Penal System,” 50.

Contemporary official sources record that the cholera epidemic affected 17% of existing prisons in the Empire in 1892, or 126 of the 718 total.^38^ 1,441 cases of infection were recorded, of which 45 per cent led to the inmate’s death. These figures most probably are understated because of deficient registration practices. However, the margin of error was not great, as the fluctuations in the overall death rate, shown in Figure 1, tend to confirm.

The most reliable measure of the impact of the cholera epidemic in prisons was excess mortality. In 1890, before the cholera outbreak prison doctors registered 4,071 deaths or 3.8% of total average prison population for that year (including exiles and the members of their families, caught in the transportation system of the Empire).^39^ In the epidemic year of 1892, the absolute number of fatalities leaped to 6,645 or 5.9% of total prison population.^40^ In 1893, prison mortality started to wane and by 1894, cholera deaths had fallen into the several hundreds across the Empire. The excess deaths cannot all be attributed to cholera because famine was affecting the Empire’s population in 1892 - prisons suffered food shortages that could lead to isolated deaths by starvation. There was also imprecision in how the cause of death was recorded. As a result, the number of cholera-induced fatalities may well have deviated from the official figures. Even allowing for inaccuracies, however, the figures indicate that the measures the Imperial government put in place allowed it to avert a full-blown catastrophe. If we factor in the highly virulent and then-incurable nature of the cholera pathogen, the insignificant state capacity of the Russian Empire, poor prison conditions in the 1890s, and the catastrophic spread of the epidemic in the free population, the crisis of 1892-1893 in prisons appears grave, but certainly less severe than it could have been.^41^

A combination of factors explains the overall success achieved in the containment of the cholera epidemics in Russia prisons at the end of the nineteenth century. One factor was the imprisonment rate in the country of 93/100,000 that was relatively small. Hence the system was more manageable (by comparison, the rate in the Russian Federation today is 335/100,000).^42^ The preventative measures put in place played a major role in containing the cholera epidemic. The decision in 1893 by the Main Prison Administration director to halt exile and prisoner transportations passing through key forwarding prisons to Siberia was crucial. These efforts were aided by the development of the new communication technology of the telegraph that allowed the rapid exchange of information among St. Petersburg and the localities and facilitated nationwide coordination. At the facility level, the imposition of two-week periods of quarantine for prisoners suspected of contracting cholera and the prohibition of outsiders’ visits helped to prevent the movement of the disease through prisons.

On the therapeutic side, the state invested in additional emergency hospitals located along the Great Exile Tract to Siberia, and it increased medical supplies to transit prisons. Other measures built upon prior improvements in hygiene, sanitation, and diet associated with prison reforms informed by Howard’s medical model of the modern penitentiary. Thus, sanitation and hygiene were tightened up and prisoners’ clothes, wash houses, and latrines were disinfected with carbolic acid (*karbolka*, phenol), the anti-germ properties of which had only recently been discovered. Mercuric chloride (*sulema*), vitriol solution, burning sulphur, quicklime, and formalin were also used. Access of prisoners to bathhouses, steam cleaning of their clothes and incineration of soiled garments, and the introduction of strict regimes for boiling water and milk supplemented these measures. The Russian Orthodox Church rallied behind the efforts to quell the disease by permitting prisoners to break the Lenten fast to support their health.^43^ For all its relative backwardness, Russia had an up-to-date understanding of disease transmission upon which the prison authorities were prepared to act. In so doing, the epidemic of 1892-1893 was a valuable learning experience and set the standard for the management of subsequent epidemics.

## Reporting on Epidemics in Late Imperial Russia, 1880-1917

The relative success of containing the cholera epidemic did not mean that all premature deaths were avoided. Alongside stories of successful interventions, there were also stories of mismanagement, haphazard decision-making, and of truculent, callous attitudes of Imperial functionaries at all levels. As the cholera epidemic took hold across the Empire, the penal system was subject to bruising criticism in the two-volume book, *Siberia and the Exile System*, authored by the American explorer-journalist George Kennan.^44^ The book reported the staggering death rates in Imperial Russian prisons. Kennan did not have to bribe a bureaucrat to get these figures, but simply extracted them from easily accessible, official reports available in the public domain.

Scholars of penality in late Tsarist era can, in fact, rely on meticulously detailed statistics on mortality and sickness during epidemics in prisons produced by the GTU from the early 1880s, including down to provincial level. The detail of these data is striking by Soviet and contemporary Russian standards.^45^ The pre-revolutionary press and civil society actively criticized the disastrous situation in Imperial prisons. Public intellectuals, the international community, and investigative journalists all joined in the debate about prisoners’ health.^46^ Intense public criticism stimulated both governmental and civic action to alleviate the crisis, the latter, for example, through charity donations. By the time of the next serious epidemic in Russia’s prisons – the typhus outbreak of 1908-1910 – the GTU had clearly lost control of the dialogue about the impact of the prison and exile system it oversaw on prisoners’ health.

The publication of factual material that supported criticism of Imperial Russia’s penal policy appears to be paradoxical. True, after the 1905 revolution, the stirrings of democratisation began to put limits on the autocrat’s power but, had it wished to, the GTU could have censored the information it placed in the public arena. The free flow of information most likely reflected the struggle between liberal sections of the bureaucracy, which had for years been pushing for penal reform, and powerful conservative forces. From 1879 onwards, at least some elements in the Russian government genuinely attempted to integrate its ill-reputed prison system into the emergent transnational movement of penal reform and modernization. Reform in 1879 that centralised the administration of punishment in the GTU contributed to progressive changes, which contributed to a decrease in prison mortality rates that were sustained until Revolution and the Russo-Japanese war in 1905-1907. Russian delegates were also active participants in the International Penitentiary Congresses, the fourth of which took place in St. Petersburg in 1890.^47^ The reformist drive encompassed relatively new concepts of prison personnel accountability and civilian control over the penitentiary system. The effect of these changes should not be exaggerated. The late Russian Empire remained an autocracy where the rule of law and civilian oversight over prisons were only beginning to take root. Some pre-revolutionary officials certainly resisted criticism of the penal system. Nevertheless, epidemics in prisons and the failures of efforts to contain them were not state secrets. This relative transparency was not to outlive the 1917 Revolution.

## The Typhus Epidemics of 1932-1933 and 1937-1938 in the Gulag

Typhus, an acute infectious disease, is transmitted by the human body louse.^48^ The case-fatality rate (CFR) easily reaches 40% without treatment. Importantly, typhus must not be confused with typhoid fever which is caused by a different type of bacterium. Historically, both illnesses were endemic to most prison systems, typhus earning the popular name of “gaol fever.”^49^ It thrives particularly in overcrowded conditions; the worse the congestion in a prison, the higher the typhus-induced mortality rate. In the decade before WWI and the Bolshevik revolution, typhus had raged in Russian prisons, exacerbated by rising prisoner numbers following the brutal suppression of the 1905-1907 Revolution and the loss of the Sakhalin penal colony to the Japanese in the Russo-Japanese war.^50^ In the early 1930s, another typhus epidemic threatened the captive population, but in a political and penological context radically different from the Tsarist predecessor.

On 1 January 1932, there were 266,000 prisoners in the Gulag. A massive surge in extrajudicial and judicial convictions increased that number to over one million by May 1933. The years that followed witnessed a rise in the prisoner population at an unprecedented scale and tempo.^51^ The overcrowding of facilities combined with famine, a consequence of the collectivization of agriculture, created conditions for typhus to take hold. As in pre-revolutionary times, the perpetual circulation of large numbers of prisoners between penal destinations spread the contagion through the penal estate and into the general population.^52^ In a telegram to the Council of People’s Commissars in Moscow, the chair of the Siberian region executive committee, Fedor Pavlovich Griadinskii, warned of the danger of the prison transport:

In the echelons of prisoners passing via Novosibirsk, twenty percent have body lice. There are cases of typhus. Part is seriously emaciated which hinders sanitary processing. [On] 2-3 April, 29 are ill with suspected typhus and 14 corpses were offloaded from the three train echelons. From echelon № 500 Bataisk over the course of transportation, 43 dead bodies were offloaded …There is a danger of dissemination of the diseases along the route. (5 April 1933).^53^

Griadinski’s anxiety resonated with Moscow authorities. They received hundreds of similar panicked memos from all over the country. The localities all bemoaned the same thing - irresponsible transportation of infected prisoners spreads disease in the camp-industrial complex via railroads. The prospect of mass typhus-induced deaths was a cause for concern for the central authorities because of its potential impact on the size of Gulag labor force needed for the frenzied industrialization drive of the first five-year plan. Genrikh Yagoda, the chief of the secret police (the OGPU-NKVD), tasked prison personnel to quell the typhus epidemic in the Gulag. In April 1933, he insisted in a report to Stalin that typhus was under control in the labor camps: “There were no epidemic diseases, in particular typhus, in the OGPU camps, except for single cases…Since there are large typhus epidemics in the detention houses of almost all the regions of the USSR, typhus was brought into the OGPU camps with the new replenishment, which we managed to stop everywhere, preventing it from spreading to the camp prisoners.”^54^

This quote lucidly exemplifies a typical blame-deflection strategy, used by secret police in these years. Yagoda relegated responsibility for an epidemic emergency to the rival bureaucracy of the Commissariat of Justice in charge of civilian prisons. The latter ostensibly sent infected prisoners into the OGPU camps and vitiated the Gulag’s otherwise impeccable sanitary conditions. However, as Tables 1 and 2 show, Yagoda’s assurance that no infected prisoners were allowed to enter labor camps was incorrect. The deficit of qualified medical personnel and rudimentary diagnostic techniques meant that the typhus epidemic was already deeply embedded both in the camps and detention houses.

**Table 1.** The number of typhus cases in the GULAG camps, 1933-1934

Camps		Jan	Feb	Mar	Apr	May	Jun	Jul	Aug	Sep	Oct	Nov	Dec	TOTAL
**1.Bamlag**	**1933**	0	45	69	26	153	73	7	1	31	8	6	17	**436**
	**1934**	8	4	6	7	2	3	1	2	6	2	5	22	**68**
**2.Dmitlag**	**1933**	8	5	13	39	80	16	6	0	11	17	2	9	**206**
	**1934**	9	9	8	11	10	4	0	4	4	0	0	1	**60**
**3.BBK**	**1933**	1	24	23	50	14	1	0	0	0	0	13	5	**131**
	**1934**	48	26	7	1	1	0	1	1	1	2	2	1	**91**
**4.UkhtPechLag**	**1933**	0	0	3	6	9	5	0	0	1	1	0	0	**25**
	**1934**										2			**2**
**5.Svir’lag**	**1933**	4	0	2	29	15	8	0	2	0	0	0	1	**61**
	**1934**		2	6		2	1							**11**
**6.Temlag**	**1933**	0	28	27	40	4	3	0	0	0	0	4	0	**106**
	**1934**											1		**37**
**7.Slag**	**1933**			0	15	1	0	0	0	0	0	0		**16**
	**1934**													
**8. Dal’lag**	**1933**	1	72	54	61	48	19	13	3	0	2	17	2	**292**
	**1934**	6	20	5	17	4	3	-	-		1	8	0	**64**
**9.Siblag**	**1933**	36	16	63	80	345	166	33	5	10	43	13	56	**866**
	**1934**	63	67	9	9	11	4	3	1	-	1	8	41	**217**
**10.Sazlag**	**1933**	17	0	34	21	8	23	0	0	0	0	0		**18**
	**1934**	20	7	2	18	0								**0**
**11.Karlag**	**1933**	39	13	25	63	83	16	3	2	1	4	163	56	**468**
	**1934**	14	2	4	0	1		2				6	12	**42**
**12.Vishlag**	**1933**	2	29	15	78	55	59	14	0	0	0	9	1	**262**
	**1934**	1	1	1	0	0								**3**
**13.Prorvlag**	**1933**													
	**1934**	1	0	0	1	0	1							**4**
**14.Balakhlag**	**1933**													**46**
	**1934**													
**15.Sarlag**	**1933**									1				**1**
	**1934**													
**TOTAL**	**1933**	108	266	332	510	815	389	76	130	55	76	228	169	**3,037**
	**1934**	137	170	28	64	31	16	7	8	13	9	39	77	**656**

Source: GARF.F.R-9414.Op.1.D.2740.L.12.

**Table 2.** List of instances when local police sent prisoners who were infected with typhus to jails, Western Siberia, the first half of 1932 (fragment)

Names of local police stations	Name of detention houses	Number of admitted prisoners	Number of the Ill	Notes
1.Pokrovskii and Aleiskii	Barnaul’skii	60	23	Admitted on June 19, medical check revealed the sick (mostly emaciated)
2.Topkinskii	Kemerovskii	10	1	Admitted on May 17, ill with typhus
3.Vassinkii	Omskii	1	1	Ill with typhus

Source: State Archive of Novosibirskaia oblast’ (GANO).F.R-47.Op.5.D.163.L.7

Imperfect as the data are, they indicated the trend. Official camp medical records give 869 cases of typhus in 1932, growing to 3,037 in the famine-stricken year of 1933. In Siblag, a large labor camp complex in Western Siberia, 866 prisoners were infected, accounting for 28.5% of the total camp population.^55^ The camp was a major hub in the prisoner transportation system receiving emaciated and sick prisoners from Ukraine, Northern Caucuses, and Kazakhstan, where both typhus and typhoid were rampant, which helps to account for the high infection rate. Mortality statistics for Siblag were inflated by the practice of convoy troops offloading dead and infected prisoners from transports into Siblag’s jurisdiction.

Behind the façade of denial, measures were put in place to contain the epidemic. In January 1933, Semen Grigor’ievich Firin, deputy Gulag director, approved the recommendations of Gulag doctors of what should be done. Moscow reminded officials in the regions that they would be punished for failing to follow central guidelines for combatting the epidemic.^56^ The preventative measures have a familiar ring about them from the 1908-1912 epidemic, but there were innovations. These included enhanced sanitary processing of all new arrivals, disinfection of their clothes, the institution of three-week quarantine and isolation of escapees after their re-capture, limits on the mobility of camp personnel, and prohibition of the internal relocation of prisoners, for example, to a new barrack within a camp. Special administrative boards in the provinces (*troikas and piaterka*s) were to oversee these measures. Mass mortality was not prevented, however. The death toll of Gulag inmates leapt from 4.8% of the total Gulag population in 1932 to 15% in 1933, with every sixth prisoner dying during the twelve-month period. In 1933, typhus and famine together caused premature deaths of at least 70,000 prisoners, to which must be added the deaths of 151,651 so-called special settlers, peasants deported from their homelands to remote places in the Urals, Central Asia, Far East, and the North during the collectivization drive.^57^ These grim data were strictly classified until the limited opening of archives in 1989-1991.

The 1932-1933 epidemic was not the last outbreak of typhus in the Gulag. There was another typhus epidemic in the penal system in 1937-1938, corresponding with the crisis years of the Great Terror that marked the apex of political repression in the Stalinist epoch. From July 1937 to November 1938, the secret police murdered at least 700,000 people (at a rate of roughly 1,000-1200 executions per day) and another several hundred thousand were sent to the camps under a simplified quasi-judicial procedure.^58^ The arrival of new prisoners over such a short period precipitated extreme overcrowding leading to another major outbreak in the Gulag.^59^

The memoir of former prisoner A. Shalganov gives a graphic window onto the typhus and dysentery epidemic in Bamlag, the camp in the Far East providing labor for the construction of a railway line north of Lake Baikal from Irkutsk to the Amur river:

In the dugout … something horrid was going on. People lay crowded on the bunks, under the bunks, right in the corridors. It was impossible to move without stepping on someone’s hand. The stench was terrible…[In] February, mass mortality began to occur in the camp. …Thirsty people collected handfuls of snow covered in bloody stains and put it their mouths. The weakened could not reach the latrine and defecated right there, near the dugouts. Dysentery raged with might and main. At first, 18-20 people per day died. Then 50-70. I made coffins: for two, for eight people. Then we ran out of timber. We built a barn for the corpses; the mountain grew and grew and soon rose two meters above the ground.^60^

Shalganov’s somber description is corroborated by official documents. Thus, Bamlag procurator Dimakov in a memo dated 18 February 1938 depicted a similar disastrous situation in camp hospitals and barracks, where ill prisoners were, “lying naked on long bunks, literally packed like sardines in a barrel…” According to the prosecutor, “people become brutalised, and some are nearly insane.”^61^

The epidemic was brought under control using the familiar methods of prevention and containment. At the personal request of USSR Prosecutor Andrei Ianuar’evich Vyshinskii, the strident regime of quarantine and mass disinfection was applied throughout the penal estate.^62^ Nevertheless, according to the internal, and most probably understated NKVD data, 126,585 prisoners died of starvation and diseases in 1938 alone.^63^ Once again, the simple fact of the existence of this epidemic in prisons, let alone its disastrous mortality rates, remained unknown to broader Soviet society until 1991.

## The Regime of Secrecy Around Epidemics and Mortality Statistics in Soviet Prisons

Long before the typhus epidemics in the Gulag, the relative transparency of the late Imperial prison service about prisoner health had given way to the paradigm of secrecy. October 1917 had signaled a shift in official attitudes towards unrestricted publication of data on epidemics in prisons. Soon after the Revolution, statistics on morbidity, mortality, and the results of epidemiological studies in prisons became classified information. Effectively, this removed prison epidemics from public discussion. Thus, in September 1925 the newly established censorship agency, *Glavlit* (Main Administration for Literary and Publishing Affairs), issued a top-secret list of information that was considered “secret and not subject to distribution, in order to protect the political and economic interests of the USSR.” Among other things, the list unequivocally prohibited publication of any data on the “sanitary state of the places of confinement.”^64^ Another instruction from 1930 forbade newspapers from publishing “negative information on conditions in places of detention.”^65^ Henceforth, any mentions of concentration camps, as they were then named, were subject to censorship by the secret police. Scurvy, emaciation, typhus, and dysentery, all symptoms of the dark underbelly of prison life in the 1920s and early 1930s, became a state secret.

The state’s reluctance to allow prison health data into the public domain was prompted by internal reports that described an acute situation in the camps and prisons. For example, the notorious Solovetskii camp, located on an archipelago in the White Sea, was hit by a particularly devastating typhus epidemic in 1927-1929. A classified report of the governmental commission set up under OGPU official Alexander Mikhailovich Shanin, which inspected the camp in the spring of 1930, offers a snapshot of the epidemic’s scale and consequences:

We must consider the extremely high morbidity and mortality of prisoners, largely because of the cruelty of the regime and harsh material conditions. For two quarters of 1929/30 25,552 people fell ill in hospitals, i.e. 44.6% of the population […] 3,583 people died in the same six months, i.e. 6.8% of the population, or 14% of inpatients; out of this number, 1004 people, or 26%, died of typhus and 396, or 11%, from emaciation and anaemia.^66^

Not a single statistic from this memorandum entered the public domain. Meanwhile, the system did everything it could to prevent released prisoners from disclosing information about their conditions of detention by making them sign non-disclosure agreements. Nina Fominichna Odolinskaia described one such agreements in her memoir of the Gulag: “I, such and such, leaving PO Box No. 385, certify to the administration of the PO Box that I have no complaints about it. I undertake not to disclose the information received at PO No. 385, and I sign for it.”^67^

The authorities fastidiously constructed the public image of putatively corrective labor in the Gulag as arduous, but nevertheless fair, humane, and rehabilitative. Extreme death rates and epidemic diseases were simply incommensurate with this myth. During a short period in the early 1930s, corrective-labor camps involved in the construction of major civil projects, such as the White Sea-Baltic Sea Canal (*BelBaltLag*) and the Moscow-Volga canal (*Dmitlag*), were in the spotlight of public and even international attention. The government employed these ambitious industrial projects in state-sponsored international propaganda campaigns that were accompanied by official publications glorifying the achievements of socialist economy through the “reforging” of delinquents into productive Soviet citizens. What would now be described as a coffee table book, *Belomor: An Account of the Construction of the New Canal Between the White Sea and the Baltic Sea*, represents the most famous example of such propaganda from this era.^68^ Needless to say, the book made no mention of the deadly epidemics that affected prisoners during canal construction (in fact, just one accidental death was acknowledged). In the mid-to-late 1930s, when the state gradually removed the Gulag from the public view, such publications ceased to be produced.^69^

As well as keeping sensitive data on epidemics hidden from the public, camp officials also erected barriers to the truth in the system’s internal documentation.^70^ We have been able to piece together how, by using opaque euphemisms, penal functionaries at all levels camouflaged incidents of illness and mortality in the already classified documents of the system. For example, secret police order № 045 of 29 April 1935 listed a set of codes for reporting diseases. These were to be used by commanders of convoys tasked with transporting newly convicted prisoners from pre-trial detention into the Gulag camps.^71^ In the top-secret addendum, № 6 to the order, typhoid fever acquired the cryptic label of “the first number”; typhus, “the second number”; smallpox, “the third”; cholera, “the fourth”; scurvy, “the fifth”; recurring fever, “the sixth.” The Ministry of the Internal Affairs (the NKVD-MVD) headquarters in Moscow, which administered the camps, provided instructions to the localities advising how to interpret the coded maladies, supplementing the guidelines with concrete examples to help the local officials. A telegram message from officer Seleznev read, “25 May Sverdlovsk. Three are resting number one.” The order explained that this should be deciphered as: “25^th^ May. Three sick with typhus fever were offloaded in Sverdlovsk.” In another example, a message from officer Nikitin reading “27 February Samara. Sent 3 with the second number,” was to be deciphered as “three prisoners died from typhus in Samara.” Sometimes the Gulag cryptic language acquired bizarre forms such as in Privolzhlag camp in the 1940s, where doctors concealed prisoners’ deaths under somewhat sinister collocation “black cases” (*chernye sluchai*).^72^ Gradually, the internal ciphers for various epidemic diseases become so abstruse that they perplexed even Gulag administrators. Eventually, the labels were standardized. On 7 June 1942, Deputy Director Georgii Dobrynin issued a circular № 42/234984, which established a new iteration of the codes (See Table 3).

**Table 3.** Codes for infectious diseases from GULAG directive № 42/234984, 7 June 1942

Ciphers	Infectious diseases
The first	Yellow fever
The second	Anthrax
The third	Epidemic encephalitis
The fourth	Smallpox
The fifth	Typhus
The sixth	Tularemia
The seventh	Plague
The eighth	Recurring fever
The ninth	Glanders
The tenth	Typhoid fever
The eleventh	Japanese encephalitis
The twelfth	Taiga encephalitis

Source: GARF.F.R-9414.Op.1.D.2771.L.60-60ob.

The most widespread epidemic in the Gulag was not acute infectious diseases but rather starvation. Statistically, the probability of a prisoner dying from lack of food, at least in the 1930s and 1940s, was greater than death from typhus. Gulag officials went to considerable lengths to conceal this fact, just as it had epidemics of infectious diseases. For example, on 28 May 1941 Gulag Director Viktor Grigor’ievich Nasedkin expressed his concern in a telegraphed directive №31/593372 to all camp commanders and local NKVD chiefs: “There is evidence that some sanitary departments of camps and colonies (OITK) quite often put emaciation (*istoszhenie*) as the cause of death on the death certificates. Such documents not only end up in the courts that sentenced the prisoner, but also come into the possession of prisoners’ relatives, which, in turn, provokes undesirable conclusions about the actual reason of death.”^73^ He continued to instruct camp commanders on how to disguise deaths due to starvation in reports to external organs and the civil registration system. Nasedkin’s order reminds us of how politicized, distorted, and unreliable the civil registration of prisoners’ causes of death actually was in Stalin’s times despite its secrecy.

The policy of concealing epidemics from unwelcome gaze persisted well into the post-Stalin period. As a result, for decades the fact of epidemiological emergencies in Soviet prisons was suppressed or denied outright. Memoirs and interviews with survivors of correctional labor colonies in the three decades after Stalin’s death and the partial dismantling of Gulag camps confirm that epidemics did habitually happen right up to the end of the USSR. However, only the vaguest details were ever released into the public domain, and they remain undisclosed today pending the declassification of late Soviet prison archives.

## TB-HIV Epidemic in the Post-Soviet Prisons of the 1990s

The collapse of the USSR in 1991 presented the Russian successor state with new challenges in dealing with health issues in its prisons. As we saw above, humanitarian concerns about the health and welfare of the prisoners were not a priority for the Soviet authorities. The Russian Federation’s membership of the Council of Europe (1996) and ratification of the European Convention on Human Rights (1998) changed this, bringing prisoners’ rights to the surface in all policy decision-making. Henceforth, whatever practical measures the Russian Federation’s prison service might take to control epidemics in its facilities, these would have to be shown to be consistent with respect for prisoners’ rights. Together with new international treaty obligations post-1991, the democratic transition to which the new leadership now committed the country required a degree of transparency that had been palpably missing in the previous seventy years. The epidemic of multi drug-resistant tuberculosis (MDR-TB) that spread like wildfire through prisons and into the general population in the 1990s and early 2000s illustrates how the country tried to meet this challenge as it underwent radical regime change.

Conditions in 1990s Russia created a perfect storm for the reversal of the steps that had, albeit belatedly, helped the USSR control tuberculosis in the previous decades. Economic collapse and the disintegration of the health system now combined to spread the disease as crime levels soared, briefly taking the Russian Federation to the top of the world rankings for the rate of imprisonment. Remand prisons in the metropolitan centers filled up with people drawn from the growing population of alcoholics, the homeless, mentally ill, and drug addicted. These prisoners, the majority already with compromised health, were crammed together in poorly ventilated and disintegrating cells for 24 hours a day. Food was in short supply in the 1990s; prisoners had to share bunks or sleep on the floor, temperatures swung between extremes and prison medical services barely existed. Tuberculosis thrived. Just like their forebears, prisoners were transported in packed railcars to correctional colonies in the geographic peripheries, spreading the infection across the vast prison estate. When they were finally delivered to their destination correctional colony, prisoners were assigned, as they had been for the previous six decades, to communal dormitories where they lived cheek-by-jowl with other prisoners, eating, sleeping, exercising, laboring, and falling ill together. At every stage of the journey from arrest to release, prisoners were exposed to tuberculosis. Infection rates in prisons at the epidemic’s peak topped 4,000 per 100,000 prisoners, and in some regions, the rate reached a staggering 7,000 per 100,000.

**Figure 2.**
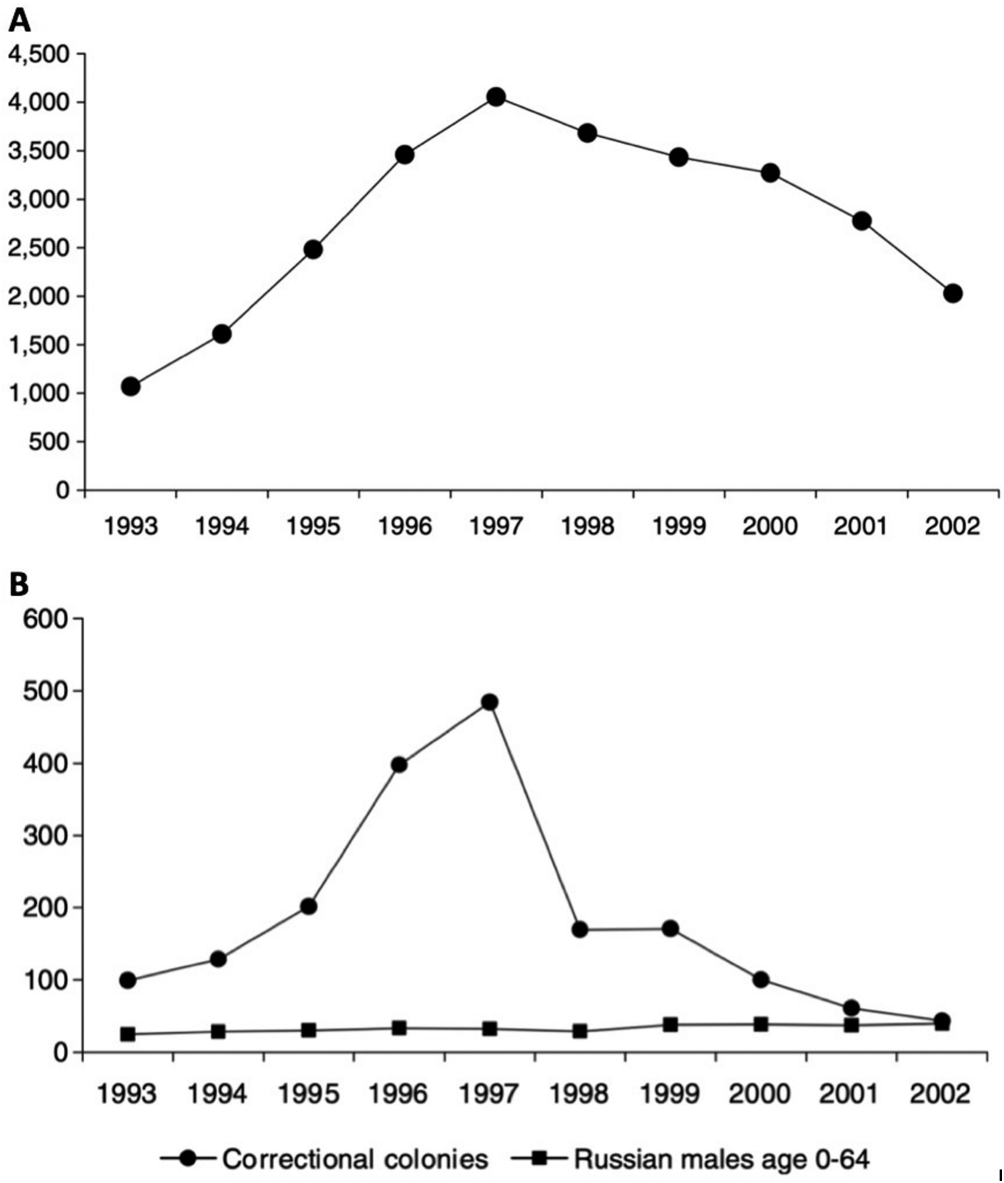
Tuberculosis in Russian Prisons 1993-2002: A: Rate (per 100,000 prisoners) of Tuberculosis infection in prisons; B: Mortality rate (per 100,000 population) prisoner and male civilian population compared.

Source: Alexey Bobrik, Kirill Danishevski, Ksenia Eroshina, and Martin McKee, “Prison Health in Russia: The Larger Picture,” *Journal of Public Health Policy* 26 (2005): 30-59. The figures for these graphs were provided to the authors by the Ministry of Justice and are not available in the public domain.

As in the Soviet period, the Prison Service in post-Soviet Russia has its own health service, separate from the civilian Ministry of Health. In the 1990s, the service was just not up to responding to the tuberculosis epidemic. The consequence was the death of many prisoners. The most vulnerable were the quarter of the total who contracted the multi-drug resistant strain of the disease.^74^ By the time the disease began to be brought under control in the 2000s, thousands of inmates had died from tuberculosis at a rate that was far higher than in the population at large.^75^

If conditions in remand prisons and correctional colonies encouraged the spread of tuberculosis throughout the prison estate, the release of thousands of infected prisoners without a post-prison treatment plan guaranteed that the disease spread into the wider community. Behind bars, prisoners with TB had to take medication but once released they would stop, either because they felt better or because they had no access to prescriptions. New press freedoms in the first decade after 1990 meant that the health crisis in prisons could no longer be hidden from either the domestic or international audience. The former communist countries were identified as the epidemiological pump for the resurgence of tuberculosis across Eurasia. One study used longitudinal analysis to examine whether, and to what degree, post-1989/1991 incarceration rates accounted for differences in TB and multidrug-resistant TB burdens among the general population in different countries of the post-communist world.^76^ The study found that the expansion of prison populations accounted for a 20.5% increase in incidence of the disease or nearly three-fifths of the average total in the decade after communism’s collapse. More controversially, it laid the blame for the resurgence of the disease on International Monetary fund (IMF) rescue packages for neoliberal economic reform.

Rather as China was identified as the source of the COVID-19 pandemic in 2020, the post-communist countries of East Central Europe and the former Soviet Union were identified in the 1990s as a MDR-TB pandemic threat to the industrialized countries of Europe and the US. Newspaper headlines warned of the “threat from the East” of a TB epidemic. Scandinavian countries were in the front line of defense when a spike in cases of MDR-TB in Sweden was traced to Russian migrants. The World Health Organization’s scientific officer stationed in Sweden warned that the threat was not just to the Scandinavian countries, but that “the elevated risk has to apply to all Western European states and to other low-incident countries like the USA.”^77^ The health panic in neighboring countries faded, but in 2014, the WHO returned to the threat, again pinpointing migration from Russia as among potential risk factors for the spread of MDR-TB in Europe.^78^

In the first decade of the new millennium, the Russian Federation managed to bring the tuberculosis epidemic more under control in the country. It was able to do this largely because of collaboration with the WHO in modernizing the country’s tuberculosis diagnostics and treatments, and financial support from the World Bank. The past two decades have seen the decline from the high infection rates in the 1990s and early years of the millennium.^79^

This decline has been mirrored in the prison estate, which the prison service attributes to investment in tuberculosis detection among prisoners newly arrived at facilities and in the building of specially purposed medical-isolation correctional colonies. More recently, the easing of overcrowding by reducing the overall number of prisoners and the repair and new builds of detention facilities also have played a role. However, a custodial sentence still means that a prisoner will almost inevitably contract the disease. One 2017 study has shown that every tenth prisoner in the Russian Federation has an active form of tuberculosis, and most prisoners carry it in latent form. MDR-TB kills more than 2000 prisoners a year, and prisoners with active tuberculosis coming to the end of a sentence still circulate the disease back into the community. Meanwhile, in a reverse movement, the ever-growing numbers of intravenous drug-users arriving in prisons are especially vulnerable to tuberculosis. Co-infection of HIV-AIDS with tuberculosis is now the primary killer of prisoners testing positive for HIV. These unwelcome developments have been accompanied by a return to Soviet-era censorship of any negative news about the state of prison health care services. Prison health statistics released into the public domain are sparse, unreliable, and obscure of the sort such as, “[I]n the last five years the number of deaths in prisons has declined by 30%, tuberculosis by 38.5%.” They are presented with no reference to absolute numbers and no disaggregation to regional or sub-regional level.^80^ Meanwhile, the volume of complaints from Russian prisoners to the European Court of Human Rights concerning prison health care - lack of hygiene, limited supplies of medications, outdated equipment and shortages of specialists, and insufficient capacity for infected prisoners in tuberculosis isolation colonies (of which there are sixty-one) – were evidence of the continuing vulnerability of prisoners to infectious disease. Russia’s expulsion from the Council of Europe in the aftermath of the country’s invasion of Ukraine does not bode well for health care reporting by the prison service in the future.

The metaphor of the “bear trap,” or its English equivalent, the “double whammy,” has been used to describe the way that both tuberculosis and HIV-AIDS have been forced together in the Russian prison, with lethal consequence for the trap’s “permanent residents.”^81^ In the early part of 2020, a new virus fell into the trap in the form of COVID-19. On 25 June 2020, Aleksandr Khabarov, deputy director of the Federal Penitentiary Service of the Russian Federation, debriefed journalists on the COVID-19 pandemic in the country’s penal institutions. He lauded the efforts of his subordinates in combating the threat of infection and praised his agency for averting a full-scale disaster. To substantiate his point, he provided data showing that more prison personnel had tested positive for the virus than prisoners.^82^ This was one in a series of similar press releases on the unfolding of the new disease behind bars that have appeared at irregular intervals. The prison service’s storyline has been consistent - that the principal health risk in prisons is not to inmates, but rather to personnel who are on the front-line of protecting the public against the spread of the virus.

At the time of writing just six deaths of prisoners, out Russia’s total incarcerated population of c. 400,000, had occurred between the arrival of the pandemic in Russia and 1 September 2021.^83^ In the unlikely event of this figure being correct, the question it raises is at what cost this extraordinary success has been achieved. The question of the cost of containment of epidemics in closed or total institutions is relevant to other jurisdictions, regardless of their position on the democratic-to-authoritarian political spectrum, and at different times. In all jurisdictions, the question can only be answered if there is transparency in reporting.

The reporting of the COVID-19 pandemic shows that old habits die slowly and the same appears to be is true of the prison service’s approach to containing the disease’s spread. Following the well-established preventative strategy, quarantine is the principal means for combatting COVID-19 in prisons. The prohibition or restriction of visits from relatives, human rights monitors, and defense counsel serves the dual purpose of keeping the disease out and unwelcome news about it in. This is all of a piece with practices refined over a century of prison epidemics.

## Conclusion

Prisons are a universal form of residential institution in which there is a fine balance to be struck between security (of the residents and of society at large) and the human rights of the people confined within, including their right to health and, ultimately, to life itself. Other such institutions historically include care homes for the elderly, psychiatric hospitals, boarding schools, poor houses, borstals, prison hulks and convict transports, and others. Prisoners are intrinsically understood as the least deserving of the people occupying society’s margins and if, to borrow from Fedor Dostoevsky, the degree of civilization in a society is to be judged by entering its prisons, the management of epidemics is one such indicator. It is true that in authoritarian countries often the analyst must wait until records become available, which may be delayed until radical change in the political regime. Precisely because of this, the study of health in prisons in authoritarian-type regimes almost always must be historical. Prison historians have tended to assume the singularity of the Russian and Soviet experience in a transnational context, but we believe there is unexplored potential for comparative studies of state responses to epidemics in other penal jurisdictions, which would situate the Russian Imperial/Soviet case more firmly in the global history of prison medicine. As our analysis has shown, prisoners’ rights have historically been the first casualty of any crisis in Russia’s prisons, but it is unlikely that this is unique to Russia.^84^ There is much scope in the historical analysis of approaches to the management of epidemics in prisons to go beyond the conventional questions of morbidity and mortality, to consider the broader impacts on human rights and also on a range of other issues such as already vulnerable family relationships, the mental health of prisoners, levels of violence, and re-offending.

Beyond the confines of the prison, pandemics and epidemics are a reminder that the functioning of the criminal justice system historically has been a public health issue and, in some countries, remains so today. The Russian case shows that prisons can emerge as epicenters of disease, but they do not have to. Just as John Howard observed in the conditions of eighteenth-century Britain, prisons do not have to be associated with poor health; it all depends upon the conditions in which prisoners are detained, which in turn, is a matter of numbers, resourcing, and prison design. The Russian example shows that the choices a society makes about how to punish offenders have an impact on the offenders’ health; cramming large numbers of people into overcrowded multiple-occupancy cells in transit and dispersal centers provides an ideal environment for the incubation of disease, while the subsequent mixing in prison transports as they were dispersed over a wide area ensures that disease spread. Russia provides a warning of the consequences of mass incarceration and its attendant warehousing of offenders on the health of vulnerable and marginal populations.

As for the future of prisoners’ health in Russia, our investigation of epidemics over the long twentieth century has shown that any meaningful future reform of the Russian prison system should start with a sharp break from Soviet-like reticence in reporting and managing epidemics. The new practices should include broad engagement of prison authorities with civil society and regular, detailed publication of qualitative and quantitative data about all registered diseases and measures that prison administration take to combat the proliferation of pathogens. Ideally, these data should not only be made freely available to the public but also monitored, re-checked, and supervised by independent medical non-governmental organizations (NGOs) created specifically for this purpose. Sadly, the prospect of changes such as these taking place in the future has retreated with the Russian invasion of Ukraine and the Russian Federation’s expulsion from the Council of Europe. It therefore falls to students of other jurisdictions to follow up on some of the questions that our study of the history of epidemics in Russian prisons has raised.

## Footnotes

1 “Rech Nachal’nika Glavnogo T’iuremnogo Upravlenia v Gosudarstvennoi Dume,” in *T’iuremnii vestnik*, No. 3 (Sankt-Peterburg: Izdanie GTU, March 1909), 282.

2 “Raziasnenia, Sdelannye Nachal’nikom Glavnogo T’iuremnogo Upravlenia v Gosudarstennoi Dume pri Obsuzhdenii Smety Ministerstva Iustit’sii po T’iuremnoi chasti 1 i 2 marta 1910 g,” in *T’iuremnii vestnik*, No. 3 (Sankt-Peterburg: Izdanie GTU, March 1910), 299.

3 The concept of the prison’s birth is associated with Michel Foucault’s influential *Discipline and Punish: The Birth of the Prison* (London: Allen Lane, 1977). For Imperial Russia the concept has been explored in Bruce F. Adams, *The Politics of Punishment: Prison Reform in Russia, 1863–1917* (DeKalb, IL: Northern Illinois University Press, 2019); Larisa Kangaspuro, “The Influence of the Revolutionary Changes on Crime and Punishment in Russia,” *Vestnik Sankt-Peterburgskogo universiteta, Istoria* 63 (2018): 763-770.

4 While communal labor and collectivism in the penal environment were certainly not uniquely Soviet or Stalinist phenomena, the distortion of these principles stemmed from the novel brutalizing emphasis on fulfilling production plans at a tacitly accepted cost of over-exploiting prisoners to death on an industrial scale. On the Gulag as a phenomenon, see Alan Barenberg and Emily Johnson, ed., *Rethinking the Gulag: Sources, Identities, Legacies* (Bloomington: Indiana University Press, 2022); Michael David-Fox, ed., *The Soviet Gulag: Evidence, Interpretation, and Comparison* (Pittsburgh: Pittsburgh University Press, 2016); Anne Applebaum, *Gulag: A History* (New York: Anchor Books, 2004); Oleg V. Khlevniuk, *The History of the Gulag: From Collectivization to the Great Terror* (New Haven: Yale University Press, 2004); Steven Barnes*, Death and Redemption: The Gulag and the Shaping of Soviet Society* (Princeton: Princeton University Press, 2011); Wilson T. Bell, *Stalin’s Gulag at War: Forced Labor, Mass Death, and Soviet Victory in the Second World War* (Toronto: Toronto University Press, 2018); Alan Barenberg, *Gulag Town, Company Town: Forced Labor and Its Legacy in Vorkuta* (New Haven: Yale University Press, 2014).

5 Judith Pallot, “The Gulag as the Crucible of Russia’s Twenty-first-century System of Punishment,” *Kritika: Explorations in Russian and Eurasian History* 16 (2015): 681-710; Laura Piacentini and Judith Pallot, “’In Exile Imprisonment’ in Russia,” *British Journal of Criminology* 54 (2014): 20-37; Laura Piacentini, and Gavin Slade, “Architecture and Attachment: Carceral Collectivism and the Problem of Prison Reform in Russia and Georgia,” *Theoretical Criminology* 19 (2015): 179-197.

6 Classic examples are Robert Conquest, *Kolyma: The Arctic Death Camps* (Oxford: Oxford University Press, 1979); Robert Conquest, *The Great Terror: Stalin’s Purge of the Thirties* (London: Macmillan, 1968).

7 Golfo Alexopoulos, *Illness and Inhumanity in Stalin’s Gulag* (New Haven: Yale University Press, 2017). See also a discussion of the issue in Alan Barenberg, Wilson T. Bell, Sean Kinnear, Steven Maddox, and Lynne Viola, “New Directions in Gulag Studies: A Roundtable Discussion,” *Canadian Slavonic Papers* 59 (2017): 376–395.

8 Philip Smith, *Punishment and Culture* (Chicago: University of Chicago Press, 2008).

9 Ibid., 27-28.

10 In developing this argument, Smith acknowledges his debt to the semiotician Mikhail Bakhtin’s concept of dialogism between centre and periphery. See Mikhail Bakhtin, *The Dialogic Imagination: Four Essays*, vol. 1 (Austin: University of Texas Press, 2010).

11 In the Gulag, for example, an internal memo stated that “A prisoner is prohibited to divulge anything about the camp and its production.” State Archive of the Russian Federation (hereafter GARF). F. R-9414.Op.1.D.327.L.81.

12 See for example, Michael Worboys. “Was There a Bacteriological Revolution in Late Nineteenth-century Medicine?” *Studies in History and Philosophy of Science Part C: Studies in History and Philosophy of Biological and Biomedical Sciences* 38 (2007): 20-42; Robert Bud, *Penicillin: Triumph and Tragedy* (Oxford: Oxford University Press, 2007).

13 Russian scientist Zinaida Ermol’eva independently synthesised penicillin for the Soviet military in WWII, and penicillin was first used in a Soviet hospital in 1943. The methods of production were inefficient, the purity of the drugs produced varied, and distribution was a problem because of the shortage of vials and packaging. See M. Schaeffer Conroy, *Medicines for the Soviet Masses During World War II* (Lanham: University Press of America, 2008).

14 Judith Pallot, “The Topography of Incarceration: The Spatial Continuity of Penality and the Legacy of the Gulag in Twentieth-and Twenty-First-Century Russia,” *Laboratorium. Zhurnal Sot’sialnikh Issledovanii* 7 (2015): 26-50.

15 N.G. Kuprianov, *Istoricheskii Ocherk Sostoiania Medit’siny v Tsarstvovanie Ekateriny II s Prilozheniem Kratkogo Obzora T’iuremnogo Dela, Sostavlennyi Nikolaem Kuprianovym, Starshim Vrachem Bol’nitsi Sankt-Peterburgskogo T’iuremnogo Zamka, Doctorom Medit’siny* (Sankt-Peterburg: tipografia Ia.Treia, 1873); E.R.Eighlot’tz, *T’iuremnii Vrach I Ego Pat’sienty* (Tuberkulez v T’iur’makh) (Petrograd: Tipografia Odinoch.t’iur’my, 1916); N.S. Lobas, *Katorga I Poselenie na O-ve Sakhaline* (Neskol’ko Shtrikhov Iz Zhisni Russkoi Shtrafnoi Kolonii) (Pavlograd: V.S.Lobas, 1903); P.P. Fridolin, *Opyt T'iuremno-Medit’sinskoi Statistiki: Otchet po Muzhskoi I Zhenskoi Bol’nitsam S.Peterburgskogo T'iuremnogo Zamka Za 1878* (Sankt-Peterburg: Tipografia L.V. Fomina, 1879).

16 Gur’iev, “Sanitarnoe Sostoianie Nashikh T’iurem. Izvlechenie Iz Doklada, Predstavlennogo Nachal’niku Glavnogo T’iuremnogo Upravlenia,” in *T’iuremnii vestnik*, No.11 (Sankt-Peterburg: Izdanie GTU, January 1910): 65-84. Gur’iev’s full reports could be found in GARF.F.122.Op.1.D.6063.L.9-97ob.

17 M.N. Gernet, *Istoriia tsarskoi t’iur’my: Petropavlovskaia krepos’t’, 1900-1917*, Vol. 4 (Moskva: Gosudarstvennoe Izdatel’stvo Iuridicheskoi literatury, 1962), 36, 43.

18 For an excellent exception to the rule see Sarah Badcock, “From Villains to Victims: Experiencing Illness in Siberian Exile,” *Europe-Asia Studies* 65 (2013): 1716-1736.

19 Stephen Wheatcroft, “The Crisis of the Late Tsarist Penal System,” in *Challenging Traditional Views of Russian History*, ed. Stephen Wheatcroft (Basingstoke: Palgrave Macmillan, 2002), 27-54.

20 Mary Schaeffer Conroy, “Health Care in Prisons, Labour, and Concentration Camps in Early Soviet Russia, 1918–1921,” *Europe-Asia Studies* 52 (2000): 1257-1274. On mortality in Civil War camps, see Mikhail Nakonechnyi, “The Devil is in the (Statistical) Details: Why Does the Quantitative History of Russian Civil War Incarceration Still Matter in 2021?” *Cahiers du monde russe* 63 (2022): 205-222.

21 Boris Nakhapetov, *Ocherki Istorii Sanitarnoi Sluzhby Gulaga* (Moskva: Rosspen, 2009); Oxana Ermolaeva, “Health Care, the Circulation of Medical Knowledge, and Research in the Soviet Gulag in the 1930s,” *East Central Europe* 40 (2013): 341–365; Dan Healey, “Lives in the Balance: Weak and Disabled Prisoners and the Biopolitics of the Gulag,” *Kritika: Explorations in Russian and Eurasian History* 16 (2015): 527-556.

22 Healey, “Lives in the Balance,” 529.

23 Elizar Moiseevich Meletinskii, a famous historian of culture and former Gulag prisoner, remembered an old prison doctor with a pre-revolutionary degree during his incarceration. See E.M.Meletinskii*, Izbrannye Stat’ i. Vospominaniia* (Moskva: RGGU, 1998), 497.

24 Golfo Alexopoulos, “Medical Research in Stalin’s Gulag,” *Bulletin of the History of Medicine* 90 (2016): 363-393.

25 For “six million deaths” estimate see Alexopoulos, *Illness and Inhumanity in Stalin’s Gulag*, 16, 243. On 2.5 million estimate, see Mikhail Nakonechnyi, “Factory of Invalids: Mortality, Disability, and Early Release on Medical Grounds in Gulag, 1930-1955” (PhD diss., University of Oxford, 2020), 323-326. For official (and understated) 1.7 million figure, see V.P. Kozlov, ed. *Istoriia Stalinskogo Gulaga: Konets 20– kh—Pervaia Polovina 50–kh godov. Vol.4: Chislennost’ i Uslovia Soderzhania* (Moskva: Rosspen, 2004), 55.

26 Sidney Bloch and Peter Reddaway, *Soviet Psychiatric Abuse: The Shadow Over World Psychiatry* (New York: Routledge, 2019). Valuable empirical material on material conditions in the post-Stalin Gulag is provided by Jeffrey S. Hardy, *The Gulag after Stalin: Redefining Punishment in Khrushchev’s Soviet Union* (Ithaca: Cornell University Press, 2016).

27 S.P.Mishustin, *Ispolnenie Nakazania v Lechebnikh Ispravitel’nikh Uchrezhdeniakh FSIN Rossii: Teoretiko-Pravovoi i Organizat’sionnii Aspekti* (Samara: Samarskii iuridicheskii institut FSIN Rossii, 2017); N.V.Davydova, L.F.Pertli, *Penitent’siarnaia Medit’sina: Istoriia i Liudi: Uchebnoe Posobie* (Moskva: NII FSIN Rossii, 2011).

28 Kseniia Runova, *T’iuremnaia Medit’sina: Print’sipy i Keisy dlia Rossii*, vol. 5 (Sankt-Peterburg: Institut problem pravoprimenenia, 2020); Kseniia Runova, “Between Autonomy and Dependency: Medical Professionals in the Modern Russian Penal System,” *Journal of Power Institutions in Post-Soviet Societies [Online]* 19 (2018); “Neumestnaia Gumannost: Kak Rabotaiut Vrachi v Penitents’iarnoi Systeme Rossii,” *Journal of Social Policy Studies* 17 (2019): 345-358.

29 Charlotte E. Henze, *Disease*, *Health Care and Government in Late Imperial Russia: Life and Death on the Volga, 1823-1914* (New York: Routledge, 2011).

30 A.G.Rashin, *Naselenie Rossii za 100 let (1811-1913). Statisticheskie Ocherki* (Moskva: GSI, 1956), 208.

31 G.G.Onischenko et. al, *Medit’sina I Imperatorskaia Vlast’ v Rossii: Zdorov’e Imperatorskoi Sem’i I Medit’sinskoe Obespechenie Pervikh Lit’s Rossii v XIX-nachale XX veka: Po Materialam Deiatel’nosti Prodvornoi Medit’sinskoi Chasti Ministerstva Imperatorskogo Dvora Ego Imperatorskogo Velichestva, 1 Ianvaria 1843-15 Junia 1918* (Moskva: Media Press, 2008).

32 John Davis, *Russia in the Time of Cholera: Disease under Romanovs and Soviets* (London: I.B. Tauris and Co., 2019).

33 On the number of infections and deaths from cholera, see Rashin, *Naselenie Rossii*, 208. On famine of 1891-1892, see Richard G. Robbins, *Famine in Russia, 1891-1892: The Imperial Government Responds to a Crisis* (New York: Columbia University Press, 1975).

34 Today, cholera is understood as a more complex condition than described by Snow and Koch’s postulates. For a history of the disease, see Christopher Hamlin, *Cholera: The Biography* (Oxford: Oxford University Press, 2009).

35 On exile see Sarah Badcock, *A Prison without Walls? Eastern Siberian Exile in the Last Years of Tsarism* (Oxford: Oxford University Press, 2016); Andrew Gentes, *Exile, Murder and Madness in Siberia, 1823-61* (Basingstoke: Palgrave Macmillan, 2010); Daniel Beer, *The House of the Dead: Siberian Exile under the Tsars* (London: Allen Lane, 2016); Sarah J. Young, “Knowing Russia’s Convicts: The Other in Narratives of Imprisonment and Exile of the Late Imperial Era,” *Europe-Asia Studies* 65 (2013): 1700–1715; Ben Phillips, *Siberian Exile and the Invention of Revolutionary Russia, 1825-1917: Exiles, Émigrés and the International Reception of Russian Radicalism* (London: Routledge, 2022).

36 On Galkin-Vrasskoi, see S.V. Zubov*, Mikhail Nikolaevich Galkin-Vrasskoi: Gosudarstvennaia i Obzhestvennaia Deiatel’nost* (Saratov: KUbiK, 2017).

37
*Otchet po Glavnomu T’iuremnomu Upravleniu za 1892* (Sankt-Peterburg: Izdanie GTU, 1893), 76-80.

38 The figure includes Tsarist Poland but excludes Finland. See *Otchet po Glavnomu T’iuremnomu Upravleniu za 1892* (Sankt-Peterburg: Izdanie GTU, 1893), 140.

39 Imperial penal populations consisted of two main legal types, prisoners per se and various types of exiles. Only hard-labor exile (*ssyl’nokatorzhnie*) were deprived of liberty and held in special *katorga* prisons. Other exiles were not incarcerated. However, all exiles, accompanied by their wives and children, were initially transported to their designated exile locations with prisoners proper through a sophisticated system of forwarding prisons. See A.P.Salomon, *Ssylka v Sibir’: Ocherk Ee Istorii i Sovremennogo Polozheniia: Dlia… Komis. o Meropriiatiiakh po Otmene Ssylki* (Sankt-Peterburg: Tipografia Sankt-Peterburgskoi T'iur’my, 1900).

40 See *Otchet po Glavnomu T’iuremnomu Upravleniu za 1890; za 1892; za 1893* (Sankt-Peterburg: Izdanie GTU). On transportation in a specific Russian context see Sarah Badcock and Judith Pallot, “Russia and the Soviet Union from the Nineteenth to the Twenty–First Century,” in *A Global History of Convicts and Penal Colonies*, ed. Clare Anderson (London: Bloomsbury Academic, 2018), 271–306.

41 By invoking the limited state capacity of the Russian Old Regime, we do not argue that Tsarist bureaucrats were, by and large, unqualified civil servants. On the contrary, pre-revolutionary prison officials tended to be far more educated and professionally trained than Gulag’s functionaries. However, the fundamental problem was in their insignificant quantities. Tsarist Russia was a weak, dramatically “under-governed” empire, plagued by a chronic shortage of cadre bureaucrats. Small state apparatus, albeit qualified in itself, often could not handle the tremendous burden of administrating such a huge country and its sprawling penal system. B.N. Mironov, *Rossiiskaia Imperia: Ot Tradit’sii k Modernu* (Sankt-Peterburg: Dmitrii Bulanin, 2015), 422-489.

42 For the Russian Imperial rate, see Jonathan Daly, “Criminal Punishment and Europeanization in Late Imperial Russia,” *Jahrbücher Für Geschichte Osteuropas* 48 (2000): 359. For Russian rate, see World Prison Brief, Institute for Criminal Policy Research World Prison Population List, 12th ed., November 2018.

43 On the described measures against cholera, see *Otchet po Glavnomu T’iuremnomu Upravleniu za 1892; za 1893* (Sankt-Peterburg: Izdanie GTU). For the correspondence between provincial governors and the GTU on the issue of cholera containment see GARF.F.122.Op.1.D.2390; F.122.Op.2.D.509; F.122.Op.2.D.1256.F.122.Op.6.D.1475; F.122.Op.6.D.1683; F.122.Op.6.D.1855.

44 George Kennan, *Siberia and the Exile System*, vol. 2 (Cambridge: Cambridge University Press, 2012).

45
*Sveden’ia o Boleznennosti i Smertnosti Sredi Arestantov v Mestakh Zakluchenia Grazhdanskogo Vedomstva* (Sankt-Peterburg: Izdanie GTU, 1882).

46 E.g., *Atrocities in Russian Prisons* (London: Society of Friends of Russian Freedom, 1913).

47 On 1879 reform, see Adams, *Politics of Punishment*.

48 Emmanouil Angelakis, Yassina Bechah, and Didier Raoult, “The History of Epidemic Typhus,” *Microbiology Spectrum* 4: 4 (2016).

49 In the Russian Imperial prisons, typhus and typhoid cases were meticulously recorded by doctors already in 1890. See GARF.F.122.Op.1.D.1870.L.122ob.-123.

50 At its height, the typhus epidemic in 1909 had claimed the lives of several thousand prisoners, taking the prison mortality rate to 3.8% per annum (calculated based on *Otchet po GTU za 1909*). As in the 1891-1892 cholera epidemic, the GTU had responded by ordering the assiduous screening of prisoners, quarantine, and the wide disinfection of facilities and prisoners’ clothes. By 1910, the epidemic had been brought under control. For measures against typhus see GARF.F.122. Op.6 D.2961. In 1905, the Russian government evacuated former Sakhalin hard-labour convicts (*katorzhane*) and exiles in a coordinated, large-scale extraction effort. The authorities redistributed Sakhalin exiles and convicts in prisons and exile localities of the Russian Far East, European Russia, and Siberia (notably, in historic Nerchinsk Katorga District and Alexandrovskii central prison). For more details see GARF.F.102. Op.144.5-oe deloproizvodstvo.1905. D.119L.B.L.1-96. On the general history of Sakhalin penal colony, see Andrew A. Gentes, *Russia’s Sakhalin Penal Colony, 1849-1917: Imperialism and Exile* (London: Routledge, 2021).

51 V.N.Zemskov, “Demografia zakluchennikh, spet’sposelent’sev, i ssyl’nikh (30-e-50-e gody),” *Mir Rossii. Sot’siologia. Etnologia* 8 (1999): 114; Oleg Khlevniuk, *Khoziain: Stalin i Utverzhdenie Stalinskoi Diktatury* (Moskva: Rosspen, 2010), 167.

52 For example, see Siberian materials in State Archive of Novosibirskaia oblast’ (hereafter GANO). F.P-47.Op.5.D.163.L.7. See also Vladimir Isupov, “Epidemii Sypnogo Tifa Kak Faktor Sverkhsmertnosti Gorodskogo Naseleniia Zapadnoi Sibiri (1932 – 1933 gg.),” *Ural´skii Istoricheskii Vestnik* 44 (2014): 90-95.

53 GANO.F.P-47.Op.5.D.169.L.107.

54 Istoriia Stalinskogo Gulaga Vol. 3, *Ekonomika Gulaga*, 95.

55 GARF.F.R-9414.Op.1.D.2740.L.12.

56 Istoriia Stalinskogo Gulaga, Vol. 4, 467-469.

57 Ibid., 465, 477. On special settlements, see Lynne Viola, *The Unknown Gulag: The Lost World of Stalin’s Special Settlements* (Oxford: Oxford University Press, 2007).

58 V.P.Danilov, R.Manning, L.Viola, ed., *Tragedia Sovetskoi Derevni. Kollektivizat’sia i Raskulachivanie. 1927-1939*, Vol. 5, B.2. (Moskva: Rosspen, 2006), 304.

59 Khlevniuk, *Khoziain*, 369-370.

60 Quoted in S.A. Papkov, *Stalinskii Terror v Sibiri, 1928-1941* (Novosibirsk: Izdatel’stvo Sibirskogo Otdelenia Rossiskoi Akademii Nauk, 1997), 132.

61 Khlevniuk. *History of the Gulag*, 174-175.

62 Istoriia Stalinskogo Gulaga, Vol. 4, 483.

63 Ibid., 55.

64 A.Blium, *Za Kulisami Ministerstva Pravdy: Tainaia Istoria Sovet’skoi Tsenzury, 1917-1929* (SPB: Akademicheskii Proekt, 1994), 125-126.

65 G.M. Ivanova, *Istoriia Gulaga, 1918–1958: Sotsial´no–ėkonomicheskii i Politiko–pravovoi aspekty* (Moskva: Nauka, 2006), 21.

66 Istoriia Stalinskogo Gulaga, Vol. 4, 146.

67 The euphemism “PO box” signified a camp or colony. N.F. Odolinskaia, *Sovet’skie Katorzhanki* (Odessa: Astroprint, 1998), 136.

68 M. Pieshkov, L. Averbakh, S. Firin, and A.Williams–Ellis, *The White Sea Canal: Being an Account of the Construction of the New Canal between the White Sea and the Baltic Sea* (London: John Lane, 1935).

69 David Dallin and Boris Nicolaevskyi, in *Forced Labor in Soviet Russia* (London: Hollis and Carter, 1948), comment, “The more forced labour has developed since 1930, the denser has become the official fog that shrouded this institution in mystery” (49).

70 On secrecy in the Gulag’s medicine see Alexopoulos, *Illness and Inhumanity in Stalin’s Gulag*, 86.

71 GARF.F.R-9401.Op.1A.D.7.L.26-36.

72 GARF.F.R-9407.Op.1.D.343.L.4.

73 GARF.F.R-9414.Op.1.D.2762.L.103.

74 Alex Mercer, Bart Jacobs, S. Moon, J. Kynch, “Prisons and the Tuberculosis Epidemic in Russia,” *Journal of International Development* 15 (2003): 559-574. The development of multi-drug resistant strains is an unintended and inevitable consequence of anti-biotic (TB) drugs and incomplete treatment programmes.

75 “TB an Epidemic in Russia’s Prisons,” *Newsline*, April-May 1999, 33.

76 David Stuckler, Sanjay Basu, Martin McKee, Lawrence King, “Mass Incarceration Can Explain Population Increases in TB and Multidrug-resistant TB in European and Central Asian Countries,” *Proceedings of the National Academy of Sciences* 105 (2008):13280-13285. The study found that each percentage point increase in incarceration rates of 0.34% (population attributable risk, 95% C.I.: 0.10–0.58%, *P* < 0.01), after controlling for TB infrastructure, HIV prevalence, and several surveillance, economic, demographic, and political indicators.

77 “Russia Spreading TB, Report Says,” *Tampa Bay Times,* 25 March 1998, https://www.tampabay.com/archive/1998/03/25/russia-spreading-tb-report-says/.

78 “Tuberculosis Levels in Russia and Belarus ‘Disaster,’ WHO Says,” *Moscow Times,* 4 July 2014, https://www.themoscowtimes.com/2014/07/04/tuberculosis-levels-in-russia-and-belarus-disaster-who-says-a37025; WHO, “Global Tuberculosis Report 2014,” 22 October 2014, https://reliefweb.int/report/world/global-tuberculosis-report-2014.

79 The infection rate in the general population now hovers around 45-50 per 100 000 and since 2012, mortality has declined by nearly one half. See “Russian Federation Sees 9.4% Drop in TB Cases in 2017,” last modified April 19, 2018, https://www.who.int/europe/news/item/19-04-2018-russian-federation-sees-9-4-drop-in-tb-cases-in-2017.

80 Lyudmila Al’pern, “Rossikaya Ti‘ur’ma Poprezhnemy Opasna Dlya Zhizni,” last modified 13 July 2019, https://mbk-news.appspot.com/suzhet/rossijskaya-tyurma-po/.

81 The metaphor describes an “inescapable predicament.” F.A. Drobniewski, R. Atun, I. Fedorin, A. Bikov, and R. Coker, “The ‘Bear Trap’: The Colliding Epidemics of Tuberculosis and HIV in Russia,” *International Journal of STD & AIDS* 15 (2004): 641–646.

82 “V Ucherezhdeniiakh FSIN Rossii Udalos Izbezhat’ Sluchaev Gibeli ot Koronavirusa,” TASS News Agency, last modified 25 June 2020, https://tass.ru/obschestvo/8810335.

83 “V Rossiiskikh SIZO i T‘iur’makh s Nachala Pandemii Umerli ot Koronavirusa Vsego Shest’ Osuzhdennykh,” Interfax Rossia News Agency, last modified 19 September 2021 https://www.interfax-russia.ru/moscow/news/v-rossiyskih-sizo-i-tyurmah-s-nachala-pandemii-umerli-ot-koronavirusa-vsego-shest-osuzhdennyh. This figure is the last placed in the public domain, as of 1 June 2022.

84 In the case of the SARs COVID-19 pandemic, prisoners’ faced the following constraints: loss of right to visits from relatives and human rights monitors, loss of rights to phone and to write home, and the right to receive parcels. The latter in Russia is necessary for the observation of religious and non-standard (i.e., meat based) dietary practices. In 2022 there was a partial lifting of these restrictions, but the powers under which they were introduced remain in place and available for other emergency situations. See Olga Zeveleva and José Ignacio Nazif-Munoz, “COVID-19 and European Carcerality: Do National Prison Policies Converge When Faced with a Pandemic?” *Punishment & Society* 24 (2022): 642-666.

